# How BMP-2 induces EMT and breast cancer stemness through Rb and CD44?

**DOI:** 10.1038/s41419-017-0037-0

**Published:** 2018-01-16

**Authors:** Guanglin Zhang, Peide Huang, Anan Chen, Weiyi He, Zhen Li, Ge Liu, Ju Wang

**Affiliations:** 1 Institute of Biomedicine, Guangdong Provincial Key Laboratory of Bioengineering Medicine, National Engineering Research Centre of Genetic Medicine, College of Life Science and Technology, Jinan University, Guangzhou, 510632 China; 2 Section for Molecular Disease Biology, Department of Drug Design and Pharmacology, Faculty of Health and Medical Sciences, University of Copenhagen, 2200 Copenhagen N, Denmark; 3 Guangzhou Institutes of Biomedicine and Health, Chinese Academy of Sciences, 190 Kai Yuan Avenue, Science Park Luogang, Guangzhou, 510530 China

Tumor cells, originating from rare stem cells responsible for maintaining tumors, are organized hierarchically in certain malignancies^1^. Breast cancer stem cells (BCSCs, CD44^+^/CD24^−^) promote tumor progression and exhibit enhanced invasive properties that favor distant metastasis in patients with breast cancer^2, 3^.

In our previous study, we showed that bone morphogenetic protein (BMP)-2 inhibited cancer cell growth in vitro and in vivo by inducing G1 arrest and apoptosis in MDA-MB-231 and MCF-7 human breast cancer cell lines^4^. BMPs are known to be involved in metastatic progression and tumorigenesis of many types of cancer^5^, but functional studies have revealed contradictory roles of BMPs in both cancer promotion and inhibition^6^. Consequently, in our recent publication, we investigated the mechanism underlying the effect of BMP-2 on breast cancer metastasis using a comprehensive molecular approach in breast cancer cell lines and clinical breast cancer samples^7^.

We observed that rhBMP-2 induced epithelial–mesenchymal transition (EMT) in three breast cancer cell lines (MCF-7, MDA-MB-231, and a mouse breast cancer cell line 4T1) and enhanced the migratory and invasive capabilities of these cells both in vitro and in vivo. Next, we used the RT²Profiler PCR array (Qiagen, Hilden, Germany) to detect changes in the expression of 84 genes known to be associated with tumor metastasis. The most upregulated genes were *CD44* and *MMP11*, while the most downregulated genes were *RB1* and *CDH1* (E-cadherin)^7^.

CD44, an alternatively spliced transmembrane protein, functions as a receptor for hyaluronan and a. co-receptor for multiple receptor kinases associated with breast cancer^8^. *CD44* expression is essential for maintaining the cancer stem cell phenotype^9^. Immunocytochemistry assays showed that rhBMP-2 upregulated *CD44* expression and induced the redistribution of cellular CD44 to the leading edges and lamellipodia of MCF-7 cells. Using Smad4–siRNA silencing and the *CD44* promoter-luciferase reporter system, we further showed that rhBMP-2 upregulated *CD44* expression in MCF-7 cells via the conventional Smad-dependent signaling pathway. Binding of Smad4 to the SBE (Smad-binding element)-rich region of the *CD44* promoter activated *CD44* expression. BMP-2 also promoted the formation of tumor spheroids and increased the population of CD44^+^/CD24^−^ cells in MCF-7 breast cancer cells. These observations suggest that rhBMP-2 enhances the stemness of breast cancer cells.

Rb (retinoblastoma) is a well-known tumor suppressor that initiates and maintains cell cycle arrest and modulates apoptosis. Functional loss of the *RB* contributes to aggressive tumor phenotype and induces EMT in breast cancer^10^. Unlike the Rb Ser567 phosphorylation-mediated and p-38 signaling pathway-activated induction of ubiquitin-dependent degradation of Rb in melanoma cells^11^, we observed that Rb was phosphorylated on Ser807/811 and subjected to ubiquitin-dependent degradation through a Smad-independent PI3K/AKT signaling pathway in BMP-2-activated breast cancer cells. Thus, we identified a unique mechanism of rhBMP-2 -mediated Rb downregulation that promotes metastasis in MCF-7 cells.

Our results further showed that Rb reduction and activation of the PI3K/Akt pathway contributed partially to *CD44* upregulation. *CD44* expression was significantly upregulated in Rb-silenced cells than in control MCF-7 cells. rhBMP-2-mediated CD44 upregulation was impaired in cells pretreated with PI3K and AKT inhibitors (LY294002 and MK-2206)^7^. These results were consistent with those of a recent study, which showed that *CD44* expression was required for collective motility and metastatic progression initiated by loss of Rb function in breast cancer^12^. Our study also suggested that cross-talks between the Rb and CD44 pathways were required for BMP-2-dependent EMT and development of BCSCs.

Overall, this is the first study demonstrating that BMP-2 is a driving factor for promoting EMT and breast cancer stemness via Rb and CD44 signaling pathways (Fig. 1). Finally, we suggest that both PI3K/AKT and Smad signaling are involved in the rhBMP-2-mediated regulation of *RB* and *CD44* expression. Our in vitro and in vivo findings highlight the crucial roles of BMP-2, Rb, and CD44 in breast cancer metastasis, which may provide new strategies for determining the prognosis and treatment of advanced breast cancer.

**Fig. 1:**
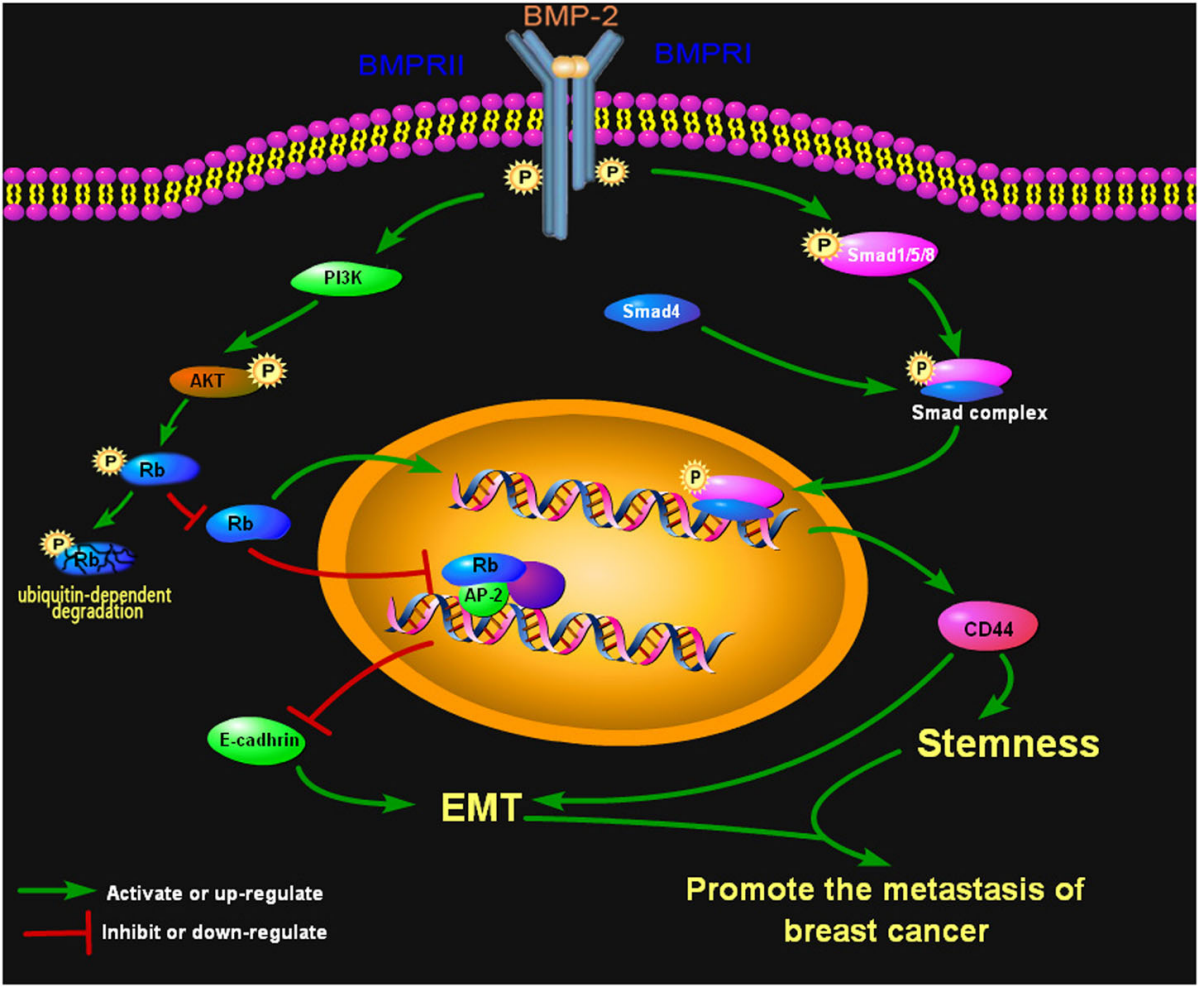
This scheme depicts the signaling pathways via which BMP-2 induces EMT and stemness of breast cancer cells through Rb and CD44 and contributes to breast cancer metastasis. The PI3K/AKT and Smad signaling pathways are implicated in the BMP-2-mediated regulation of Rb and CD44, and a crosstalk exists between Rb and CD44 signaling pathways

## References

[1] Burgosojeda D, Bo RR, Buckanovich RJ (2012). Ovarian cancer stem cell markers: prognostic and therapeutic implications. Cancer Lett..

[2] Sheridan C (2006). CD44 + /CD24 – breast cancer cells exhibit enhanced invasive properties: an early step necessary for metastasis. Breast Cancer Res..

[3] Abraham BK (2005). Prevalence of CD44+/CD24-/low cells in breast cancer may not be associated with clinical outcome but may favor distant metastasis. Clin. Cancer Res..

[4] Chen A (2012). Inhibitory effect of BMP-2 on the proliferation of breast cancer cells. Mol. Med. Rep..

[5] Kang MH, Kim JS, Ji ES, Sang CO, Yoo YA (2010). BMP2 accelerates the motility and invasiveness of gastric cancer cells via activation of the phosphatidylinositol 3-kinase (PI3K)/Akt pathway. Exp. Cell Res..

[6] Wang L, Park P, La Marca F, Than KD, Lin CY (2015). BMP-2 inhibits tumor-initiating ability in human renal cancer stem cells and induces bone formation. J. Cancer Res. Clin. Oncol..

[7] Huang P (2017). BMP-2 induces EMT and breast cancer stemness through Rb and CD44. Cell Death Discov..

[8] Ponta H, Sherman L, Herrlich PA (2003). CD44: from adhesion molecules to signalling regulators. Nat. Rev. Mol. Cell. Biol..

[9] Owens TW, Naylor MJ (2013). Breast cancer stem cells. Front. Physiol..

[10] Knudsen ES (2015). RB loss contributes to aggressive tumor phenotypes in MYC-driven triple negative breast cancer. Cell Cycle.

[11] Delston RB, Matatall KA, Sun Y, Onken MD, Harbour JW (2011). p38 phosphorylates Rb on Ser567 by a novel, cell cycle-independent mechanism that triggers Rb-Hdm2 interaction and apoptosis. Oncogene..

[12] Kim KJ (2013). Rb suppresses collective invasion, circulation and metastasis of breast cancer cells in CD44-dependent manner. PLoS ONE.

